# Predictive and discriminant validity of the Impaired Control Scale – Cannabis (ICS-C): an intensive longitudinal study of daily cannabis use outcomes

**DOI:** 10.1186/s42238-026-00441-9

**Published:** 2026-05-09

**Authors:** Erin L. Courtice, Nicolle Fox, Christian S. Hendershot, Jeffrey D. Wardell

**Affiliations:** 1 Department of Psychology, York University, Toronto, Canada; 2 Department of Population and Public Health Sciences, University of Southern California, Los Angeles, CA USA; 3 Institute for Addiction Science, University of Southern California, Los Angeles, CA USA; 4 Department of Psychiatry and the Behavioral Sciences, University of Southern California, Los Angeles, CA USA; 5 Institute for Mental Health Policy Research, Centre for Addiction and Mental Health, Toronto, Canada; 6 Department of Psychiatry, University of Toronto, Toronto, Canada

## Abstract

Impaired control over substance use – difficulty adhering to limits on consumption – is a core feature of problematic use and addiction. Although extensively studied in alcohol research, impaired control over cannabis remains understudied. To advance research in this area, we evaluated the predictive and discriminant validity of the Impaired Control Scale–Cannabis (ICS-C) using an intensive longitudinal design. Young adults (*N* = 147; *M*age = 22.07) who reported regular use of cannabis and alcohol completed three 21-day ecological momentary assessment (EMA) bursts spaced six months apart. The ICS-C was administered prior to each burst, followed by daily surveys assessing difficulty limiting use, substance consumption, and consequences. Three-level multilevel models examined whether ICS-C scores predicted cannabis outcomes observed on the daily surveys and whether daily reports of difficulty limiting cannabis use mediated these associations. The Perceived Impaired Control subscale demonstrated strong predictive validity: Within-person increases in perceived impaired control across bursts predicted greater difficulty limiting cannabis use in daily life, which in turn mediated associations with heavier cannabis consumption and use of multiple forms of cannabis. At the between-person level, the Attempted Control subscale predicted negative consequences through difficulty limiting cannabis use. The ICS-C also demonstrated discriminant validity, showing no significant associations with alcohol outcomes after controlling for cannabis use. The ICS-C, particularly the Perceived Impaired Control subscale, captures meaningful within-person fluctuations in self-regulatory capacity that predict real-world cannabis use patterns. Findings support the ICS-C as a valid tool for examining impaired control over cannabis across multiple temporal levels.

**Supplementary Information:**

The online version contains supplementary material available at 10.1186/s42238-026-00441-9.

Impaired control over substance use is a central feature of problematic use and addiction, capturing the discrepancy between intention and behaviour. Specifically, impaired control is defined as “a breakdown of an intention to limit consumption” (Heather et al. 1993, p. 701); a pattern in which substance use exceeds conscious intentions, despite efforts to restrict it (American Psychiatric Association 2022; Leeman et al. 2007; 2012; 2014). Impaired control is distinct from related risk factors such as trait impulsivity or generalized poor self-regulation. Whereas impulsivity reflects a broad predisposition toward rash action, and poor self-regulation captures difficulties managing behavior across multiple domains, impaired control describes substance-specific failures to adhere to consumption limits that individuals have set for themselves (Leeman et al. 2012, 2014). Extensive work in the alcohol literature has established impaired control over alcohol as an early-emerging and potent predictor of problematic drinking and related harm. Among young adults, greater impaired control is associated with heavier drinking, more frequent binge episodes, and higher rates of alcohol-related consequences (Leeman et al. 2009; Martínez-Loredo et al. 2020). Longitudinal research also suggests that impaired control over alcohol mediates associations between distal risk factors (e.g., impulsivity, poor self-regulation) and later alcohol problems (Patock-Peckham et al. 2018; Wardell et al. 2016). In this way, impaired control functions as a mechanism in the development of problematic use (Leeman et al. 2014).

Despite decades of work on alcohol-related impaired control, research on impaired control over cannabis use has lagged. This gap is concerning given the rising prevalence and normalization of cannabis use, particularly among young adults in regions that have legalized or decriminalized use (Hall et al. 2023). In Canada, 48% of young adults (aged 20 – 24) reported using cannabis at least once in past year (Health Canada 2024), and rates of cannabis use among young adults are similarly elevated in U.S. states with legal cannabis (Patrick et al. 2022). Although most cannabis use is non-problematic, frequent or heavy use increases risk for cannabis use disorder (CUD) (Leung et al. 2020). Emerging evidence suggests that impaired control may be a key process in the escalation from recreational to problematic cannabis use. For example, cross-sectional and longitudinal studies suggest that symptoms reflecting difficulty cutting down, using more than intended, or failing to resist use are among the most common symptoms associated with CUD onset (Pellegrino et al. 2020; van der Pol et al. 2013). Yet, compared with the extensive alcohol literature, empirical research on impaired control over cannabis remains sparse.

One reason for this gap is the limited availability of psychometrically robust measures that comprehensively capture impaired control over cannabis. By comparison, in the alcohol literature, there is a widely used and well-established measure developed by Heather et al. (1993): the Impaired Control Scale (ICS). The ICS provides a detailed assessment of several dimensions of impaired control over alcohol, including: (a) attempted control (frequency of efforts to limit use), (b) failed control (difficulty adhering to limits), and (c) perceived impaired control (beliefs about future ability to limit use). Together, these dimensions differentiate between the motivation to regulate use (attempted control) and low capacity to do so successfully (failed control and perceived impaired control). Factor-analytic work has generally supported a two-factor structure, with a separate attempted control factor and substantial overlap between failed control and perceived impaired control components (Heather et al. 1998; Marsh et al. 2002). Indeed, the failed control and perceived impaired control subscales share nearly identical content, differing only in whether the items refer to past experiences of failed control versus beliefs about future ability to control alcohol use (perceived impaired control). Consequently, several studies have focused on one or the other of these scales, depending on the research question (Leeman et al. 2012; 2024; Wardell et al. 2015; 2018). Regardless of subscales used, impaired control as measured by the ICS is associated both cross-sectionally and prospectively with heavy drinking, alcohol use disorder, and treatment outcomes across clinical and non-clinical samples (Heather and Dawe 2005), underscoring its predictive validity.

Until recently, there was no comparable measure available for assessing impaired control over cannabis. For instance, although the Marijuana Consequences Questionnaire (MACQ; Simons et al. 2012) includes a brief impaired-control subscale (the MACQ-IC), this subscale is packaged as part of a broader measure of cannabis consequences and was not intended to be used as a standalone, comprehensive assessment of the impaired control construct. Indeed, this subscale is imbalanced in its measurement of attempted control (efforts to limit consumption) versus failed control (difficulty limiting consumption) and does not include specific items tapping into perceived impaired control. To address the need for a more comprehensive measure of impaired control over cannabis, Taguba et al. (2022) developed the ICS-C by adapting items from the alcohol ICS to reflect cannabis use contexts. Their initial validation with a sample of Canadian university students identified two reliable and distinct subscales: (1) attempted control and (2) failed control. The study revealed some psychometric challenges: reverse-scored items consistently formed a separate factor (although this may reflect a broader challenge with reverse-scored items; Weijters et al. 2013), and the perceived impaired control items were not distinct from failed control (although this was expected based on the redundancy in item content). Based on these results, Taguba et al. (2022) recommended creating subscales without reverse scored items and chose to focus on the failed control items without their perceived control counterparts. These scales demonstrated strong initial evidence for convergent and discriminant validity relative to cannabis and alcohol use outcomes.

However, Taguba et al. (2022)’s study was cross-sectional and relied on retrospective self-report, which limited the ability to examine the measure’s predictive validity or assess its properties over time. Furthermore, although Taguba et al. (2022) focused on the failed control subscale, perceived impaired control may be more informative in young adult, non-treatment-seeking samples. Specifically, perceived impaired control reflects perceived capacity to regulate use even among those not actively attempting to limit use (Leeman et al. 2014), which may be helpful for predicting future impaired control in prospective studies. In contrast, failed control may be more situationally sensitive and most informative for assessing past experiences of impaired control in clinical or treatment contexts, where efforts to regulate use are more explicit. Thus, further validation of the ICS-C for use among young adults is needed using longitudinal designs and focusing on the perceived impaired control dimension.

## The current study

The current study extends Taguba et al.’s (2022) prior validation work on the ICS-C by evaluating its predictive validity within an intensive longitudinal design using Ecological Momentary Assessment (EMA). EMA methods capture people’s experiences in real time and in their natural environments, providing a more ecologically valid test of whether the ICS-C predicts meaningful variability in self-regulation of cannabis use in everyday life. Our data were collected across three EMA bursts spaced six months apart, with each burst consisting of baseline administration of the ICS-C, followed by daily surveys administered over 21 consecutive days to assess day-level difficulty limiting cannabis use, cannabis use behavior, and acute consequences. This design allows for an explicit test of whether impaired control as measured by the ICS-C can predict difficulty limiting cannabis use in everyday life, and an examination of how within-person changes in impaired control relate to cannabis use and problems over the course of a year. We examined both the Attempted Control (Part 1) and Perceived Impaired Control (Part 3) subscales, as prior alcohol research suggests these dimensions may relate differently to alcohol outcomes (Heather et al. 1998; Ingesson et al. 2022; Wardell et al. 2018).

There were four key research objectives guiding the current study. First, we evaluated the predictive validity of the ICS-C by testing the extent to which burst-level and person-level ICS-C scores predict day-to-day reports of difficulty limiting cannabis use in naturalistic contexts captured through EMA. We hypothesized that the Perceived Impaired Control scale would predict difficulty limiting use at both the burst level and between-person level. Second, we also examined predictive validity by investigating whether the ICS-C predicts cannabis use outcomes (quantity used, variety of products used, and negative consequences) over time, at both burst and between-person levels. We hypothesized that perceived impaired control would relate to cannabis use and problems over time. Third, we tested whether daily reports of difficulty limiting cannabis use mediate associations between the ICS-C and both cannabis use and consequences. Here, we hypothesized that the ICS-C’s relationship with cannabis outcomes would be explained by participants’ daily reports of difficulty limiting use; this finding would provide further evidence for validity by suggesting that it is the experience of impaired control *in the moment* that links ICS-C with cannabis outcomes (rather than some other process). Finally, leveraging our sample of individuals who regularly use both cannabis and alcohol, we assessed discriminant validity by comparing the strength of associations between the ICS-C and cannabis outcomes versus parallel alcohol outcomes. Here, we hypothesized that the ICS-C would not predict difficulty limiting alcohol use or alcohol-related consequences.

## Methods

### Participants and procedure

The analytic sample consisted of 147 young adults (*M*age = 22.1, *SD* = 2.1) residing in Ontario, Canada and of legal age to purchase both alcohol and cannabis. Full details of participant recruitment and EMA data collection procedures are reported in Wardell et al. (2024; 2025). Briefly, participants were recruited as part of a larger ecological momentary assessment (EMA) study on alcohol and cannabis co-use. Eligibility criteria required that participants: (a) were between 19 and 25 years of age, (b) had used both alcohol and cannabis at least once per week over the past month, (c) had engaged in simultaneous use of alcohol and cannabis at least twice in the past month, and (d) owned a smartphone compatible with the EMA app (MetricWire, Waterloo, Canada). Exclusion criteria were (a) current treatment for or attempts to reduce substance use, (b) self-reported use of cannabis exclusively for medical purposes, (c) current severe mental illness, or (d) using drugs other than cannabis, alcohol, or nicotine more than once per month. For the current analyses, we retained only participants who endorsed cannabis or alcohol use on at least one study day (*N* = 147), as analyses were limited to use days. Of these, 128 (87.1%) reported both cannabis and alcohol use during the study period, 11 (7.5%) reported cannabis use only, and 8 (5.4%) reported alcohol use only. The analytic sample for the primary cannabis models was *n* = 139 (excluding the 8 participants who reported no cannabis use), and the analytic sample for the alcohol discriminant validity models was *n* = 136 (excluding the 11 participants who reported no alcohol use). See Table 1 for characteristics of the sample used in the current analyses.

**Table 1:** Participant sociodemographic characteristics

	***N***	***%***
Sex assigned at birth		
*Female*	94	63.9
*Male*	52	35.4
Gender		
*Woman*	88	59.9
*Man*	55	37.4
*Transgender and/or nonbinary*	7	4.8
Race/Ethnicity		
*White*	91	61.9
*Asian*	23	15.6
*East Indian*	15	10.2
*Black*	13	8.8
*Hispanic/Latinx*	7	4.8
*Middle Eastern*	6	4.1
Education Level		
*High school or less*	52	35.4
*Some college*	32	21.8
*Associate degree*	8	5.4
*Bachelor’s degree*	52	35.4
*Graduate degree*	3	2.0
Employment		
*Unemployed*	46	31.3
*Employed part-time*	65	44.2
*Employed full-time*	36	24.5

*N* = 147. Percentages may not sum to 100% due to rounding. Some category totals do not sum to *N* = 147 because participants could select “prefer not to respond.” Gender identity and race/ethnicity categories were not mutually exclusive, as participants could select multiple identities

**Fig. 1:**
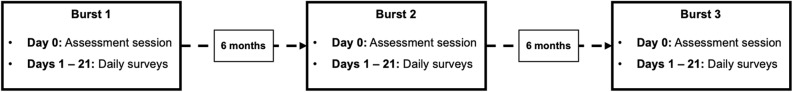
Longitudinal burst study design including three measurement bursts spaced six months apart. At each burst, participants completed a Day 0 assessment session (ICS-C administered online) followed by 21 consecutive days of daily morning surveys, which assessed previous-day cannabis and alcohol use, difficulty limiting use, and consequences experienced

After providing informed consent, participants completed a baseline session in which they were oriented to the EMA protocol and trained to report alcohol and cannabis quantities using visual aids adapted from the Daily Sessions, Frequency, Age of Onset, and Quantity of Cannabis Use Inventory (DFAQ-CU; Cuttler and Spradlin 2017). Participants were then enrolled in the EMA study, which followed a longitudinal burst design (see Fig. 1). Specifically, participants completed three 21-day EMA bursts, which were scheduled six months apart. All bursts were preceded by an assessment session during which participants completed an online survey that included the ICS-C (described below). Each burst began the day after the assessment session. The EMA protocol included several daily surveys, but the current analyses used data from only the daily morning surveys, wherein participants reported substance use and associated outcomes from the previous day. Morning surveys were delivered via the smartphone app at 7:00 AM each day, with subsequent reminder notifications until the time the survey closed at 2:00 PM. Participants received cash or gift cards worth CAD $40, $50, or $60 for participating in the baseline, 6-month, and 12-month sessions respectively, and up to $90, $105, and $120 for full study compliance across the first, second, and third 21-day EMA periods, respectively. The study protocol was approved by the Research Ethics Boards at York University and the Centre for Addiction and Mental Health, Toronto, Canada.

### Measures

#### Impaired Control Scale – Cannabis (ICS-C)

Participants completed the ICS-C (Taguba et al. 2022) during the baseline/assessment session preceding each measurement burst. This measure includes items adapted from the alcohol ICS (Heather et al. 1993), assessing (a) attempts to limit cannabis use (Attempted Control), (b) failures to adhere to limits (Failed Control), and (c) beliefs about future control (Perceived Impaired Control). We scored the ICS-C in a manner consistent with Taguba et al.’s (2022) recommendations (based on their factor analyses), which included removing the reverse-coded items. Items were rated on a five-point Likert scale and summed within subscales (see Table S1, Supplementary Material). Items comprising the Attempted Control subscale assessed attempts to control cannabis use in the past 30 days (*1* = *Never* to *5* = *Always*), and items comprising the Perceived Impaired Control subscale assessed beliefs about the inability to limit cannabis use in the future (*1* = *Strongly Disagree* to *5* = *Strongly Agree*). Both subscales demonstrated good to excellent internal consistency in the current sample. For the Attempted Control subscale: Burst 1 α = 0.90, ω = 0.90; Burst 2 α = 0.91, ω = 0.91; Burst 3 α = 0.96, ω = 0.96. For the Perceived Impaired Control subscale: Burst 1 α = 0.86, ω = 0.86; Burst 2 α = 0.86, ω = 0.86; Burst 3 α = 0.88, ω = 0.89.

#### Cannabis and alcohol use

During the EMA period, on each daily survey, participants reported whether they had used cannabis the previous day. If so, they also responded to items assessing quantities of cannabis used (grams of flower) as well as the variety of cannabis forms used (flower, concentrates, edibles). Also on each daily survey, participants were asked if they used alcohol the previous day. If so, they reported the number of standard drinks consumed.

#### Difficulty limiting cannabis and alcohol use in daily life

On each daily survey when cannabis use was reported, participants responded to a binary item assessing event-level impaired control over cannabis use, which was adapted from the Impaired Control subscale of the MACQ (Simons et al. 2012). The item read, “I found it difficult to limit how much cannabis I used yesterday” (0 = no, 1 = yes). On each daily survey when alcohol use was reported, participants responded to a parallel binary item: “I found it difficult to limit how much alcohol I drank yesterday” (adapted from the Impaired Control subscale of the Young Adult Alcohol Consequences Questionnaire; YAACQ; Read et al. 2006).

#### Cannabis and alcohol use consequences

On days when cannabis and/or alcohol use was endorsed, participants responded to 16 binary (yes/no) items that assessed acute negative consequences. These items were adapted from the MACQ and YAACQ. Thirteen of these items were not specific to cannabis or alcohol use and were administered regardless of which substance was used with a prompt asking if the consequence had occurred following “cannabis and/or alcohol use”. Three items were substance-specific (e.g., “I drove a vehicle after using [cannabis/alcohol]” and were only administered for the substance the participant had endorsed that day. If both cannabis and alcohol use were endorsed on a given day, these items were administered twice, with reference to each substance respectively. We summed the number of consequences participants endorsed to create a count variable for cannabis consequences on cannabis use days (including the non-specific and cannabis-specific items) and alcohol consequences on alcohol use days (including the non-specific and alcohol-specific items); these count variables excluded the ‘difficulty limiting cannabis/alcohol use’ items described above.

### Analytic approach

We specified a three-level multilevel structural equation model in Mplus Version 8.8 (Muthén and Muthén, 2017) to examine day-level (Level 1), burst-level (Level 2), and person-level (Level 3) associations among the ICS-C scales (attempted control and perceived impaired control), difficulty limiting cannabis use in daily life, and daily cannabis-related outcomes. Mplus uses latent variable modeling to disaggregate variance in variables across the three levels of the model. Variables measured at the day level (difficulty limiting use, negative consequences, cannabis quantity, and use of multiple cannabis forms) were specified at all three levels of the model. The ICS-C, measured at the burst level, was specified at Levels 2 and 3 of the model. All models used the Bayes estimator with 100,000 iterations and a probit link for categorical outcomes. We used uninformative priors, allowing us to interpret the 95% credible intervals (CI) as analogous to 95% confidence intervals in traditional frequentist analyses, such that 95% CIs that did not contain zero were considered statistically significant. The ICS-C scales were rescaled by dividing by 10 to improve model stability, as the sum scores have relatively high variance compared to other variables in the model, which can cause estimation problems.

The model included only observations when cannabis use occurred (*n* = 2,411 total days across 281 burst clusters from 139 participants). The model was specified to test our key research questions regarding predictive validity. At the day level (Level 1), difficulty limiting use was modelled as a binary (yes/no) outcome, negative consequences were modelled as an ordinal variable with three levels (0 consequences, 1 consequence, or 2 or more consequences), number of cannabis forms used were modelled as a binary variable (1 form or multiple forms used), and grams of cannabis flower used was specified as a continuous variable. At the burst level (Level 2), we examined whether changes in ICS-C scores across bursts predicted changes in daily reports of difficulty limiting cannabis use, as well as cannabis use quantity, number of forms of cannabis used, and negative consequences across each burst. At the between-person level (Level 3), we tested whether between-person differences in ICS-C scores were associated with between-person differences in these same outcomes aggregated across all bursts and days. To determine whether everyday experiences of difficulty limiting cannabis use captured on the daily surveys mediated the ICS-C’s influence on cannabis outcomes, we modeled difficulty limiting cannabis use as a predictor of cannabis outcomes (grams of flower used, using multiple forms of cannabis, and negative consequences) at all three levels. Indirect (mediated) paths were specified from the ICS-C to cannabis outcomes through difficulty limiting use at both the burst and between-person levels. These indirect effects were estimated using the product of coefficients approach (a-path*b-path), implemented through Mplus’ MODEL CONSTRAINT command. The statistical significance of indirect effects was evaluated using Bayesian 95% credible intervals. Day level reports of alcohol use (yes/no)^1^ were included as a covariate and specified on all three levels of the model. At the between-person level (Level 3), sex assigned at birth was included as a covariate (grand mean centered). We freely estimated random intercepts and residual covariances among outcomes at each level.

To examine discriminant validity, we specified a parallel model in which ICS-C was specified as a predictor of alcohol-related outcomes, by substituting difficulty limiting cannabis use and cannabis outcomes with their alcohol counterparts (including number of drinks and number of consequences on drinking days). The alcohol model included only observations when alcohol use occurred (*n* = 1,487 observations across 284 bursts from 136 participants). Cannabis use assessed on the daily surveys (yes/no) was included as a covariate in this model.

## Results

### Descriptive statistics

Table 2 presents descriptive statistics for key study variables across the three 21-day EMA bursts. A total of 6609 daily surveys were completed across all participants and all EMA bursts, and survey completion rates ranged from 87.5% to 92.2% across EMA bursts. ICS-C scores showed moderate stability across bursts, with approximately 50% of variance in Attempted Control and 56% of variance in Perceived Impaired Control attributable to stable between-person differences (ICCs = 0.50 and 0.56, respectively), while also demonstrating meaningful within-person variance across the three measurement occasions.

**Table 2:** Descriptive statistics for key variables

	**Baseline** **(Burst 1)**		**6-month follow-up (Burst 2)**		**12-month follow-up (Burst 3)**	
	***N***	***%***	***N***	***%***	***N***	***%***
Compliance rate	2764	89.5	1966	87.5	1879	92.2
Cannabis use days	1141	41.3	623	31.7	654	34.8
*Days using cannabis flower (vs. other forms)*	822	72.0	469	75.3	491	75.1
Days difficulty limiting cannabis use was endorsed	44	3.9	37	5.9	42	6.4
Alcohol use days	672	24.3	445	22.6	370	19.7
Days difficulty limiting alcohol use was endorsed	38	5.7	33	7.4	18	4.9
	***M***	***SD***	***M***	***SD***	***M***	***SD***
Grams of cannabis on cannabis use days	0.52	0.76	7.42	0.79	4.86	0.79
Number of standard drinks on alcohol use days	3.58	2.92	3.49	2.57	3.63	3.12
Number of negative consequences experienced on use days	0.76	1.41	0.79	1.50	3.12	0.81
Attempted Control (ICS-C Part 1) scores	9.96	4.57	11.71	5.26	11.45	5.81
Perceived Impaired Control (ICS-C Part 3) scores	12.19	5.38	12.18	5.08	11.65	5.39

*N* = 147. Compliance rate = completed surveys / expected surveys (i.e., N participants × 21 days). Cannabis and alcohol use day percentages reflect proportion of completed daily surveys. Difficulty limiting use percentages reflect proportion of respective use days. Continuous variables (grams, drinks, consequences) are averaged across relevant use days. ICS-C scales were assessed once per burst at baseline; values represent burst-level averages across participants

### Predictive validity of the ICS-C

The model demonstrated convergence, with all Potential Scale Reduction factors for model parameters below 1.01 for the final 10,000 iterations. See Table 3 for model estimates. At the day level, difficulty limiting cannabis use significantly predicted negative consequences and cannabis quantity but not using multiple forms of cannabis. As hypothesized, Perceived Impaired Control (Part 3) significantly predicted difficulty limiting cannabis use at the burst level, such that higher perceived impaired control immediately prior to a given burst was associated with reporting greater difficulty limiting cannabis use across the daily surveys in the corresponding burst. In other words, within-person changes in Perceived Impaired Control from burst-to-burst predicted changes in difficulty limiting cannabis use in daily life, supporting predictive validity of this ICS-C scale. In contrast, Attempted Control (Part 1) showed no significant association with difficulty limiting cannabis use at the burst level. Although neither ICS-C subscale showed significant direct associations with cannabis outcomes at the burst level, Perceived Impaired Control showed significant indirect associations with cannabis outcomes through difficulty limiting use (see Table 3). Specifically, higher Perceived Impaired Control at a given burst indirectly predicted heavier cannabis flower consumption and a greater number of cannabis product forms used mediated through greater difficulty limiting cannabis use.

**Table 3:** Predictive validity analyses examining the associations of the Impaired Control Scale – Cannabis with cannabis related outcomes

	**Day Level** **(Level 1)**			**Burst Level** **(Level 2)**			**Person Level** **(Level 3)**		
	Estimate	95% CIs	β	Estimate	95% CIs	β	Estimate	95% CIs	β
Direct Effects									
Cannabis Use Consequences									
*Attempted Control (ICS-C Part 1)*	–	–	–	0.33	−0.27, 0.86	0.17	−0.57	−2.50, 0.78	−0.18
*Perceived Impaired Control (ICS-C Part 3)*	–	–	–	0.06	−2.23, 2.16	0.01	1.97	−1.19, 5.03	0.22
*Difficulty Limiting Cannabis Use*	0.37*	0.23, 0.53	0.34	0.48	−0.16, 1.25	0.41	0.63*	0.03, 1.46	0.54
*Alcohol Use (yes/no)*	0.26*	0.16, 0.36	0.24	−0.40	−1.10, 0.13	−0.29	0.31	−0.24, 0.82	0.20
* Sex Assigned at Birth*	–	–	–	–	–	–	−0.06	−0.65, 0.67	−0.03
Cannabis Flower Quantity									
* Attempted Control (ICS-C Part 1)*	–	–	–	−0.00	−0.36, 0.28	−0.00	−0.03	−0.84, 1.04	−0.02
* Perceived Impaired Control (ICS-C Part 3)*	–	–	–	−0.90	−2.30, 0.20	−0.26	0.38	−1.75, 2.59	0.07
* Difficulty Limiting Cannabis Use*	0.11*	0.07, 0.16	0.26	0.42*	0.14, 0.85	0.65	−0.13	−0.69, 0.20	−0.20
* Alcohol Use (yes/no)*	0.05*	0.02, 0.08	0.11	−0.07	−0.43, 0.21	−0.09	0.14	−0.09, 0.46	0.17
* Sex Assigned at Birth*	–	–	–	–	–	–	0.20	−0.22, 0.54	0.16
Number of Cannabis Product Forms									
* Attempted Control (ICS-C Part 1)*	–	–	–	−0.71	−1.77, 0.21	−0.28	0.71	−0.81, 2.39	0.30
* Perceived Impaired Control (ICS-C Part 3)*	–	–	–	−1.88	−4.95, 1.03	−0.23	0.83	−2.33, 3.97	0.12
* Difficulty Limiting Cannabis Use*	−0.01	−0.21, 0.20	−0.01	1.31*	0.42, 2.45	0.83	−0.05	−0.82, 0.59	−0.06
* Alcohol Use (yes/no)*	0.05	−0.08, 0.18	0.05	−0.27	−1.33, 0.72	−0.14	0.35	−0.14, 0.95	0.31
* Sex Assigned at Birth*	–	–	–	–	–	–	−0.87*	−1.66, −0.22	−0.51
Difficulty Limiting Cannabis Use									
* Attempted Control (ICS-C Part 1)*	–	–	–	0.26	−0.32, 0.86	0.16	1.46*	0.54, 2.45	0.55*
* Perceived Impaired Control (ICS-C Part 3)*	–	–	–	2.27*	0.50, 4.15	0.45*	1.05	−1.82, 3.89	0.14
* Alcohol Use (yes/no)*	–	–	–	0.19	−0.44, 0.82	0.16	0.26	−0.19, 0.71	0.20
* Sex Assigned at Birth*	–	–	–	–	–	–	−0.54*	−1.09, −0.03	−0.29*
Indirect Effects									
Attempted Control (ICS-C Part 1) via Difficulty Limiting Cannabis Use									
* →Cannabis Use Consequences*	–	–	–	0.09	−0.19, 0.60	–	0.86*	0.03, 2.71	–
* →Cannabis Flower Quantity*	–	–	–	0.10	−0.14, 0.45	–	−0.16	−1.11, 0.36	–
* →Number of Cannabis Forms*	–	–	–	0.31	−0.43, 1.28	–	−0.06	−1.42, 0.94	–
Perceived Impaired Control (ICS-C Part 3) via Difficulty Limiting Cannabis Use						–			–
* →Cannabis Use Consequences*	–	–	–	0.99	−0.35, 3.19	–	0.55	−1.24, 3.20	–
* →Cannabis Flower Quantity*	–	–	–	0.92*	0.13, 2.29	–	−0.07	−1.31, 0.52	–
* →Number of Cannabis Forms*	–	–	–	2.86*	0.36, 6.20	–	−0.00	−1.30, 1.28	–

*N* = 139. Estimates represent unstandardized coefficients. At Level 1, standardized coefficients (β) are reported only for continuous outcomes. ICS-C Part 1 and Part 3 scores were rescaled by dividing by 10. Number of cannabis product forms represents the total count of different cannabis types used (0 = flower only, 1 = flower plus one other form, 2 = flower plus two or more other forms). * *p* < .001

*CI* Bayesian credible interval, *ICS-C* Impaired Control Scale – Cannabis

At the between-person level, Attempted Control significantly predicted difficulty limiting cannabis use, but Perceived Impaired Control did not, such that those who had higher scores on the Attempted Control scale overall (aggregated across bursts) also reported more consistent difficulty limiting cannabis use on the daily surveys across bursts. Consequently, Attempted Control showed a significant indirect association with negative consequences through difficulty limiting cannabis use (see Table 3). Specifically, those reporting greater attempts to control use on the ICS-C on average across time experienced more negative consequences overall across bursts, mediated through greater difficulty limiting use in daily life. There were no significant indirect associations between Attempted Control and either cannabis quantity or cannabis product forms at the between-person level. Perceived Impaired Control showed no significant direct (see Table 3) or indirect associations with any outcomes at the between-person level.

### Discriminant validity

The discriminant validity model also demonstrated convergence, with all Potential Scale Reduction factors at or below 1.01 for the final 10,000 iterations. See Table 4 for model estimates. Neither ICS-C subscale significantly predicted difficulty limiting alcohol use at the burst- or person-levels. Consistent with hypotheses, both ICS-C subscales showed minimal direct or indirect associations with alcohol outcomes on drinking days after controlling for day-level cannabis use, providing evidence for discriminant validity.

**Table 4:** Discriminant validity analyses examining the associations of the Impaired Control Scale – Cannabis with alcohol related outcomes

	**Within-Person Level** **(Level 1)**			**Between-Burst Level** **(Level 2)**			**Between-Person Level** **(Level 3)**		
	Estimate	95% CIs	β	Estimate	95% CIs	β	Estimate	95% CIs	β
Direct Effects									
Alcohol Use Consequences									
* Attempted Control (ICS-C Part 1)*	–	–	–	0.12	−0.65, 0.81	0.08	−0.17	−1.52, 0.90	−0.06
* Perceived Impaired Control (ICS-C Part 3)*	–	–	–	0.31	−2.84, 3.22	0.06	1.70	−1.46, 4.65	0.19
* Difficulty Limiting Alcohol Use*	0.70*	0.48 0.95	0.55	0.30	−1.52, 2.09	0.20	0.65*	0.24, 1.25	0.61
* Cannabis Use (yes/no)*	0.81*	0.51 1.14	0.24	0.73	−1.30, 2.49	0.25	0.61	−0.55, 1.98	0.16
* Sex Assigned at Birth*	–	–	–	–	–	–	0.01	−0.52, 0.63	0.00
Alcohol Quantity									
* Attempted Control (ICS-C Part 1)*	–	–	–	−0.50	−5.76, 4.20	−0.18	−1.10	−4.11, 0.64	−0.26
* Perceived Impaired Control (ICS-C Part 3)*	–	–	–	−0.92	−29.72, 27.65	−0.09	−1.84	−9.98, 4.27	−0.15
* Difficulty Limiting Alcohol Use*	1.13*	0.88, 1.37	0.49	1.77	−15.32, 16.11	0.72	0.99*	0.32, 2.64	0.67
* Cannabis Use (yes/no)*	0.25	−0.23, 0.72	0.04	−1.74	−14.66, 11.18	−0.32	1.46	−0.41, 4.65	0.28
* Sex Assigned at Birth*	–	–	–	–	–	–	1.15*	0.31, 2.46	0.37
Difficulty Limiting Alcohol Use									
* Attempted Control (ICS-C Part 1)*	–	–	–	0.24	−0.28, 0.80	0.26	0.95	−0.10, 2.04	0.33
* Perceived Impaired Control (ICS-C Part 3)*	–	–	–	1.74	−0.30, 3.94	0.52	2.39	−0.57, 5.31	0.29
* Cannabis Use (yes/no)*	–	–	–	0.57	−0.97, 2.26	0.32	−0.86	−2.14, 0.35	−0.24
* Sex Assigned at Birth*	–	–	–	–	–	–	−0.56	−1.15, 0.01	−0.27
Indirect Effects									
Attempted Control (ICS-C Part 1) via Difficulty Limiting Alcohol Use									
* →Alcohol Use Consequences*	–	–	–	0.04	−0.47, 0.81	–	0.57	−0.06 1.78	–
* →Alcohol Quantity*	–	–	–	0.24	−4.32, 5.61	–	0.88	−0.08 3.82	–
Perceived Impaired Control (ICS-C Part 3) via Difficulty Limiting Alcohol Use									
* →Alcohol Use Consequences*	–	–	–	0.40	−2.23 3.82	–	1.45	−0.35 4.49	–
* →Alcohol Quantity*	–	–	–	2.46	−25.74 31.45	–	2.19	−0.48 9.48	–

*N* = 136. Estimates represent unstandardized coefficients. At Level 1, standardized coefficients (β) are reported only for continuous outcomes. ICS-C Part 1 and Part 3 scores were rescaled by dividing by 10. * p < .001

*CI* Bayesian credible interval, *ICS-C* Impaired Control Scale – Cannabis

## Discussion

The current study provided the first longitudinal validation of the ICS-C using data from young adults who reported frequent cannabis and alcohol use. Building on Taguba et al.’s (2022) initial cross-sectional validation, we leveraged data from a longitudinal study with an EMA burst design to establish temporal associations between the ICS-C and subsequent cannabis use and consequences in daily life. Specifically, we examined whether ICS-C scores predict real-world cannabis outcomes reported on subsequent daily surveys, whether within-person changes in ICS-C scores over time correspond to changes in cannabis outcomes, and whether these associations are mediated by day-to-day experiences of difficulty limiting cannabis use. We also investigated discriminant validity by testing whether the ICS-C specifically predicts cannabis-related – rather than alcohol-related – outcomes. Overall, findings supported the predictive and discriminant validity of the ICS-C, particularly for the Perceived Impaired Control subscale, while also revealing important nuances in how impaired control operates across different temporal levels and cannabis outcomes.

### Predictive validity of the ICS-C

Consistent with hypotheses, the Perceived Impaired Control subscale demonstrated strong predictive validity at the burst level. Burst-to-burst changes in perceived impaired control predicted corresponding changes in participants’ daily reports of impaired control over cannabis, which in turn mediated associations with heavier cannabis consumption and likelihood of using multiple forms of cannabis. This pattern suggests that the ICS-C captures meaningful within-person fluctuations in perceived self-regulatory capacity that manifest in observable difficulties controlling use in everyday contexts. These within-person associations were specific to Perceived Impaired Control; Attempted Control showed no significant associations with cannabis outcomes at the burst level. This differential pattern aligns with prior work suggesting that perceived *inability* to control use (rather than attempts to control use) represents the core feature of impaired control that most proximally predicts consumption patterns (Heather et al. 1998; Taguba et al. 2022). The mediation through difficulty limiting use further suggests that self-perceptions of impaired control assessed via the ICS-C translate into regulatory difficulties in daily life, which are linked with heavier cannabis consumption as indicated by using more grams of cannabis flower and multiple forms of cannabis in each day.

These findings align with longitudinal alcohol research demonstrating that impaired control, as measured by the ICS, prospectively predicts heavier alcohol use and related problems over time (Leeman et al. 2009; Read et al. 2007). Our results extend this literature in a few ways. First, we demonstrate similar predictive validity for cannabis-specific impaired control, supporting the transdiagnostic relevance of the impaired control construct across substances. Second, using an intensive longitudinal design with multiple measurement bursts, we show that these associations operate dynamically within-person (burst-to-burst fluctuations) and between-person – temporal dynamics that have rarely been examined in prior alcohol research, which has relied primarily on single follow-up assessments over longer time intervals (e.g., freshman to senior year; Leeman et al. 2009). Third, this finding extends prior cross-sectional validation work (Taguba et al. 2022) by demonstrating that these processes unfold dynamically over time and can be observed in participants’ natural environments through EMA.

Although day-level reports of difficulty limiting cannabis use predicted more negative consequences at the day level, this association was not significant at the burst level, and thus perceived impaired control did not show significant indirect associations with consequences at the burst level, contrary to expectations. This pattern likely reflects a design limitation of the current study: the brief EMA windows at each burst (3 weeks) may not have provided sufficient time to capture the accumulation of negative consequences during a period of heightened impaired control. Additional research measuring consequences over longer time periods is needed to more fully establish the ICS-C’s predictive validity for cannabis-related harms.

At the between-person level, a different pattern emerged: Attempted Control significantly predicted negative consequences through difficulty limiting use, such that individuals who reported more attempts to control their cannabis use across bursts experienced more consequences overall. This finding may suggest that users who experience more consequences are more likely to recognize the need to attempt control (Heather et al. 1993), creating a positive association between attempts to control and consequences at the between-person level. However, this association did not manifest within-person over time (i.e., at the burst level), suggesting that the relationship between attempted control and consequences reflects stable individual differences rather than dynamic temporal processes. Notably, Perceived Impaired Control showed no significant person-level indirect effects, despite showing robust burst-level effects. This suggests that the Perceived Impaired Control subscale may be particularly sensitive to within-person changes in self-regulatory capacity across time periods. However, this between-person indirect effect of Attempted Control on consequences through difficulty limiting use did not fully replicate in a sensitivity analysis using alcohol quantity (rather than binary alcohol use) as a covariate, suggesting this association may be sensitive to model specification and should be interpreted cautiously.

Our differential findings for Attempted versus Perceived Impaired Control both converge with and extend prior alcohol research. Consistent with the broader literature, we found that perceived inability to control use (rather than mere attempts to limit use) was the stronger predictor of consumption patterns (Heather et al. 1998; Taguba et al. 2022). However, our study is among the first to examine Attempted Control alongside Perceived Impaired Control in a longitudinal design. Most prior longitudinal studies of the alcohol ICS have focused exclusively on the Perceived Control (Part 3) or Failed Control (Part 2) subscales, rarely incorporating Attempted Control (Part 1) into analyses (Leeman et al. 2012, 2014). This is a notable gap, as some research has found that Attempted Control was a stronger predictor of alcohol self-administration than Failed Control in a laboratory setting among nondependent drinkers (Vaughan et al. 2019). Our null burst-level findings for Attempted Control suggest this subscale may be less sensitive to temporal fluctuations in cannabis use behavior, instead capturing more stable individual differences in recognition of the need for self-regulation – a hypothesis that warrants further investigation. Together, these findings suggest that the two ICS-C subscales capture different aspects of impaired control: Attempted Control may reflect chronic regulatory efforts among individuals experiencing cannabis-related problems, whereas fluctuations in perceived capability to adhere to limits (Perceived Impaired Control) may be more predictive of temporal changes in use behavior.

We also found strong evidence for discriminant validity: neither ICS-C subscale predicted difficulty limiting alcohol use or alcohol-related outcomes after controlling for cannabis use, demonstrating substance-specificity of the measure. This finding is critical for establishing that the ICS-C captures impaired control over cannabis use specifically, rather than generalized self-regulatory difficulties or a tendency toward heavy substance use more broadly. The discriminant validity findings replicate and extend Taguba et al.’s (2022) cross-sectional findings in a longitudinal context, supporting the use of substance-specific impaired control measures and suggesting that self-regulatory processes may operate somewhat independently across different substances, even among individuals who use both regularly. This pattern is consistent with the broader impaired control literature demonstrating that impaired control is substance-specific rather than reflecting general impulsivity or poor self-regulation (Heather et al. 1993; Leeman et al. 2012).

### Limitations and future directions

This study is not without limitations. First, our sample consisted of non-treatment seeking young adults who used both alcohol and cannabis regularly, limiting generalizability. The ICS-C’s performance in those who are less cannabis- or alcohol-involved and in clinical populations requires further examination. Second, day-level impaired control was assessed with a single binary item focused on difficulty limiting use, and we did not assess whether participants actually attempted to limit their use on that day; future EMA studies could more comprehensively capture all aspects assessed by the ICS-C (attempted control, failed control, perceived impaired control) on the daily surveys. Third, cannabis quantity was assessed only for flower (cannabis grams), not for other cannabis forms like concentrates or edibles, which may have limited our ability to capture total cannabis consumption on days when non-flower products were used. Future research should examine whether the ICS-C predicts clinically significant outcomes such as development of cannabis use disorder, treatment seeking, or quit attempts.

## Conclusion

The current study provides robust evidence for the predictive validity of the ICS-C, particularly the Perceived Impaired Control subscale, in capturing within-person fluctuations in self-regulatory capacity that manifest as observable difficulties limiting cannabis use in daily life and predict heavier consumption. The findings advance understanding of impaired control over cannabis as a dynamic process that operates across multiple temporal levels, with distinct pathways to consumption versus consequences. Like findings in the alcohol literature where impaired control has been identified as one of the earliest-developing signs of problem drinking (Leeman et al. 2014), the ICS-C may prove valuable for early identification and intervention with young adults showing signs of emerging cannabis-related impairment. Thus, the ICS-C represents a promising tool for research on cannabis use trajectories and may prove valuable for identifying individuals at elevated risk for problematic use.

## Supplementary Information

Supplementary Material 1.

## Data Availability

The datasets generated and analyzed during the current study are not publicly available, but are available from the corresponding author (Jeffrey D. Wardell, [jwardell@yorku.ca] (mailto:jwardell@yorku.ca)) upon reasonable request, which may be subject to ethics review.

## Footnotes

1 As a sensitivity analysis, we tested a model in which the binary alcohol use covariate (yes/no) was replaced with alcohol quantity (number of standard drinks, winsorized at the 95th percentile). Within-person and burst-level findings—including all paths involving the ICS-C – did not differ substantially across model specifications. However, at the between-person level, the indirect effect of Attempted Control on negative consequences through difficulty limiting use was significant in the binary alcohol model (Est = 0.86, *p* = .021, 95% CI [0.03, 2.71]) but not in the alcohol quantity model (Est = 0.79, *p* = .031, 95% CI [-0.05, 2.66]). However, this difference reflected changes in the path from difficulty limiting use to consequences rather than paths involving the ICS-C itself.
