# Supplementary material for: Predictive and discriminant validity of the Impaired Control Scale – Cannabis (ICS-C): an intensive longitudinal study of daily cannabis use outcomes
Source: J Cannabis Res. 2026 May 9;8:79. doi: 10.1186/s42238-026-00441-9 (PMC13326297; doi:10.1186/s42238-026-00441-9)
Supplement: Supplementary file 1 — Supplementary Material 1. [file 42238_2026_441_MOESM1_ESM.docx]

**Table S1**

*ICS-C Scale Items*

| **Attempted Control (Part 1)** |
| --- |
| 1. I have tried to limit the amount of cannabis I used. |
| 1. I have tried to resist the opportunity to start using cannabis. |
| 1. I have tried to slow down my cannabis use. |
| 1. I have tried to cut down on my cannabis use (i.e., use less). |
| 1. I have tried to stop using cannabis for a period of time. |
| **Perceived Impaired Control** |
| 1. I would find it difficult to limit the amount of cannabis I used. |
| 1. I would start using cannabis even after deciding not to. |
| 1. Even when I intended to use only a small amount of cannabis in a given day, I would end up using much more. |
| 1. I would still use cannabis at times when I knew it would cause me problems (e.g., problems at work/school, with family/friends, or with the police, etc.). |
| 1. I would have an irresistible urge to continue using cannabis once I started (i.e., after a small amount, I want more). |
| 1. I would find it difficult to resist using cannabis, even for a single day. |
